# When Choice Isn’t a Choice: The Practical and Ethical Risks of the Terminally Ill Adults Bill of England and Wales

**DOI:** 10.7759/cureus.88413

**Published:** 2025-07-21

**Authors:** Hassan Ahmed

**Affiliations:** 1 Internal Medicine, Queen Elizabeth Hospital King's Lynn, King's Lynn, GBR

**Keywords:** assisted dying, autonomy, euthnasia, healthcare inequality, palliative care

## Abstract

The Terminally Ill Adults (End of Life) Bill, recently passed at the House of Commons, aims to legalise assisted dying for terminally ill adults with six months or less to live in England and Wales. While framed as a compassionate reform promoting autonomy and dignity, legalising assisted dying amidst widespread underfunding of palliative care risks institutionalising inequity and pressuring vulnerable patients towards premature death. The systemic failures, ranging from inconsistent end-of-life care access to limited psychological and logistical safeguards, undermine the notion of true choice. Moreover, assisted dying introduces moral strain for clinicians, potential biases in eligibility decisions, and long-term risks of legislative expansion. Until universal, high-quality palliative care is guaranteed, the bill may function less as a humane option and more as a symptom of an overstretched, under-resourced health system failing its most vulnerable.

## Editorial

In a historic move, the House of Commons has passed the *Terminally Ill Adults (End of Life) Bill* at its Third Reading, paving the way for assisted dying to become a legal option in England and Wales. Framed as a compassionate and regulated choice for terminally ill adults with six months or less to live, the bill promises autonomy, dignity, and relief from suffering. But from the perspective of a National Health Service (NHS) doctor, this legislation, though well-intentioned, raises serious ethical, clinical, and practical concerns that must not be ignored.

In a health system under severe strain, the introduction of assisted dying legislation cannot be separated from the financial and operational reality of care delivery. End-of-life support is labour-intensive: patients often require multidisciplinary management, symptom control, emotional support, and around-the-clock care. These services are inconsistently available across the UK and are underfunded in many regions [1]. Legalising assisted dying in this context introduces a cost-saving pathway into a system already struggling to provide equitable care to the living. A 2017 Canadian study estimated millions in healthcare savings by reducing the duration and complexity of terminal care following the legalisation of medical assistance in dying [2]. Although economic motives are not cited in the bill, systemic incentives must be examined, especially in a financially fragile NHS.

This concern is further amplified by the current crisis in palliative care. Despite the 2022 Health and Care Act mandating Integrated Care Boards (ICBs) to commission palliative services, hospices remain heavily dependent on public donations. Two-thirds of adult hospice income and up to 80% for children’s services must now be raised through charitable means [3]. In parliamentary debates, MPs from across the political spectrum have labelled this model “unsustainable,” with some warning of a growing “postcode lottery” in access to dignified end-of-life care [3]. When palliative support becomes a matter of geography and fundraising, the introduction of a legal alternative to living through that suffering cannot be seen as a neutral choice.

This creates a disturbing moral asymmetry. If dying with adequate care and dignity is not a universal guarantee, the assisted dying route, though framed as voluntary, risks becoming a default option. Patients denied hospice beds or facing inadequate pain control may come to see death as their only relief. In such cases, choosing assisted dying reflects not freedom, but desperation. The bill implicitly assumes that a patient’s decision to die is made in a context of full support, whereas in reality, that support is often missing.

Another concern is the psychological burden this law may place on patients. While legally voluntary, the existence of an assisted dying option may lead patients, especially those who are elderly, disabled, or socially isolated, to internalise pressure. They may come to believe that choosing death is more responsible, less burdensome to family, or even more rational. This moral pressure cannot be legislated away. It emerges quietly from social cues, medical tone, or unspoken expectations. It risks turning autonomy into obligation.

The introduction of assisted dying also reshapes the doctor-patient relationship. While protections for conscientious objectors exist, the cultural shift within medicine is profound. Clinicians enter the profession to alleviate suffering and preserve life. Being asked to counsel patients on how to end their lives, or to witness their deaths, may cause moral injury, especially to those working in specialities like geriatrics, oncology, and palliative medicine. The act of facilitating death is not a minor procedural adjustment; it redefines the role of the physician in a way that could undermine trust. Patients might begin to question whether their doctors value the continuation of their life, or whether death is now an accepted, even encouraged, clinical outcome. Moreover, it gives excessive decision-making power to doctors. Even with legal and procedural boundaries, clinicians serve as gatekeepers in this process. They are the ones who assess eligibility, verify suffering, and provide or deny access. This authority, especially without national consistency in training, experience, or risk tolerance, opens the door to subjective judgments and unconscious biases. Cases in the U.S. and U.K. have shown that decisions like “Do Not Resuscitate” orders are disproportionately applied to older patients, ethnic minorities, and those perceived as having a lower quality of life. A similar bias could affect decisions around assisted dying, even with procedural safeguards.

Assessing requests for assisted dying is also fraught with complexity. While the bill outlines safeguards requiring two independent assessments of mental capacity, freedom from coercion, and informed consent, real-world implementation is not straightforward. Terminal illness often brings cognitive impairment, depression, and fluctuating emotions. Studies have found that the desire for death in terminal patients is often transient, strongly tied to mood, anxiety, pain, and perception of dignity [4]. The assumption that a patient’s request for death is stable and fully autonomous is a dangerous oversimplification.

International experience suggests these risks are not hypothetical. While the bill’s scope is limited to those with six months to live, similar laws in Belgium and the Netherlands have expanded significantly over time. What began as a narrowly defined right for those with terminal illnesses has, in some cases, extended to patients with chronic conditions, mental illness, and even minors [5]. Although UK legislation currently prohibits such scenarios, the history of assisted dying laws elsewhere demonstrates the potential for legislative drift once the principle is accepted.

There are also broader systemic dangers. Legalising assisted dying could weaken public investment in palliative care. If assisted dying becomes a normalised alternative to complex end-of-life support, health systems may gradually reduce funding for the latter. Palliative care is expensive, time-consuming, and labour-intensive. Administering a lethal dose is not. While this is a stark comparison, it illustrates the policy risk: in resource-constrained environments, saving money can quickly eclipse any ethical concerns. In addition, the infrastructure required to implement the bill, review panels, interpreters, secure handling of lethal substances, and psychological evaluation, demands robust administrative systems. Many NHS Trusts, already struggling with staffing and service delivery, will be ill-prepared to operationalise these safeguards consistently. This increases the likelihood of inequity and procedural shortcuts, particularly in under-resourced regions.

The ethical foundation of this bill is further undermined by the government's failure to guarantee dignified end-of-life care for all. It’s clear that the government has not set aside adequate funding for palliative services, even as it pushes ahead with a bill that allows doctors to end patients' lives under strict conditions. The contradiction is stark: the state cannot afford to support people through their natural deaths but can afford to legislate a mechanism for their assisted deaths.

In conclusion, the *Terminally Ill Adults (End of Life) Bill* may appear as a compassionate response to human suffering, but in reality, it functions as a legislative solution to systemic healthcare failure. Legalising assisted dying in a country where access to palliative care is uneven, underfunded, and overly reliant on charitable support risks institutionalising despair. Until the NHS can guarantee that every patient has access to high-quality, timely, and compassionate palliative care, the introduction of assisted dying is not just premature, it is ethically indefensible. Compassion in healthcare must begin with the living, not with legislating their deaths in lieu of real support.
